# Comparative Analysis of Health, Inflammatory Markers, and Rumen Microbiota Between Mildly Lame and Healthy Cows

**DOI:** 10.3390/ani15040468

**Published:** 2025-02-07

**Authors:** Shuo Wang, Yushan Li, Runyu Wang, Jingjing Liu, Shengli Li, Erdan Wang

**Affiliations:** 1 Key Laboratory of Applied Technology on Green-Eco-Healthy Animal Husbandry of Zhejiang Province, College of Animal Science and Technology & College of Veterinary Medicine, Zhejiang Agricultural and Forestry University, Hangzhou 311300, China; 2024608032015@stu.zafu.edu.cn (Y.L.); 2023608031036@stu.zafu.edu.cn (R.W.); 2 State Key Laboratory of Animal Nutrition and Feeding, College of Animal Science and Technology, China Agricultural University, Beijing 100193, China; wsss@cau.edu.cn (S.W.); liujing85@cau.edu.cn (J.L.); lishengli@cau.edu.cn (S.L.)

**Keywords:** lameness, microbiota, rumen, cow, health

## Abstract

Bovine lameness leads to significant economic losses in the dairy industry. This study investigated the relationship between rumen microbiota and lameness in Holstein cows. Rumen fluid and blood samples were collected from 11 cows with mild lameness and 10 healthy cows before morning feeding. Using high-throughput sequencing and ELISA kits, we found that cows with lameness exhibited decreased rumen pH and increased lameness scores compared to healthy cows. Additionally, cows with lameness had higher blood concentrations of LPS, IL-1, IL-8, and TNF-α. Although there were no significant differences in microbial alpha diversity, principal coordinate analysis (PCoA) revealed significant differences in the rumen microbial structure between the two groups. Further analysis showed that the relative abundances of ruminal *Clostridium_IV*, *Streptococcus*, *Bacillus*, *Acinetobacter*, *Desulfobulbus*, *Methanobrevibacter*, and *Mogibacterium* were significantly higher in the lameness group, whereas *Succinivibrio*, *Lachnobacterium*, *Elusimicrobium*, *Succiniclasticum*, and *Prevotella* were significantly more abundant in the rumen of healthy cows. Importantly, the microbial interaction network in the rumen of cows with lameness was more complex, with key bacteria such as *Mogibacterium* dominating the microbial interaction network. This study highlights the relationship between rumen microbiota and lameness, providing insights into the prevention and treatment of bovine lameness.

## Simple Summary

Lameness in cows causes significant economic losses to the dairy industry and has attracted considerable attention from dairy farmers. This study aimed to explore the differences in blood inflammatory markers and ruminal microbiota between 11 mildly lame cows and 10 healthy cows. The results showed that there were significant differences in the ruminal microbiota composition between healthy and mildly lame cows, and some differential microbes were associated with blood inflammatory markers. These findings highlight the connection between the ruminal microbiota and lameness, providing insights for prevention and treatment strategies.

## 1. Introduction

Bovine lameness is a significant health issue prevalent in dairy farming worldwide, particularly among high-yielding dairy cows [1]. Lameness not only causes mobility challenges and reduced productivity but also increases veterinary treatment costs and can lead to premature culling in severe cases [2]. In the United States, economic losses due to lameness in dairy cows are estimated to range from USD 120 to USD 330 per cow annually [3]. Clinically, lameness is often characterized by hoof swelling, inflammation, pain, and deformities, with severe cases exhibiting pronounced limping. Additionally, lameness may lead to secondary bacterial infections, exacerbating inflammatory responses and imposing long-term adverse effects on overall bovine health [4].

Current strategies to address lameness in dairy cows mainly focus on prevention and clinical treatment. Preventive measures, such as regular hoof trimming, foot baths with disinfectants, maintaining a clean farming environment, and providing soft bedding, primarily target external factors causing hoof injuries [5,6,7]. However, these methods are less effective in managing endogenous hoof diseases, as their precise pathogenesis remains unclear. Clinical treatment approaches primarily involve pharmacological interventions and mechanical hoof trimming. Pharmacological treatments typically include antibiotics and anti-inflammatory drugs to alleviate inflammation and infections, but these are mostly symptomatic and fail to address the underlying causes [8,9]. Mechanical hoof trimming removes diseased tissues and applies antibiotics to reduce pain but provides limited resolution of the root causes [9,10,11]. With the growing issue of antibiotic resistance, the efficacy of traditional treatments is diminishing, underscoring the need for novel and effective strategies for the prevention and treatment of lameness.

In recent years, studies have shown that the rumen microbiota plays a crucial role in maintaining the digestive health and immune function of dairy cows, making it a hot topic in dairy cow health research [12]. As the core part of the digestive system, the rumen harbors a diverse and functionally varied microbial community, including bacteria, archaea, fungi, and protozoa [13,14]. These microorganisms break down complex plant fibers and ferment them to produce volatile fatty acids (such as acetic acid, propionic acid, and butyric acid), which provide an energy source for the cows [13,14]. In addition, rumen microbes are involved in protein synthesis, B-vitamin synthesis, and immune regulation [14]. The balance of the rumen microbiota is directly related to the metabolic function and overall health of the cow. However, an imbalance in the rumen microbiota (dysbiosis) can lead to a range of health problems, such as ruminal acidosis, metabolic disorders, and impaired immune function, which in turn can trigger other diseases in cows, including mastitis and ketosis [15,16]. Passos et al., after reviewing 16 related studies, pointed out that the negative impact of high-concentrate diets on the rumen microbiota might be one of the key factors in the development of lameness [17]. When cows consume high-grain diets, the proliferation of lactic acid bacteria in the rumen increases rapidly, leading to a decrease in rumen pH and resulting in ruminal acidosis [18]. This condition may trigger an inflammatory response and affect hoof health. Moreover, some studies have suggested that ruminal acidosis may cause intestinal barrier dysfunction, allowing endotoxins to enter the bloodstream, which intensifies inflammation and provides a new perspective on the development of lameness [18]. Apart from dietary structure, trace elements and fatty acids in the cow’s diet may also indirectly influence hoof health by regulating the rumen microbiota. Studies have shown that deficiencies in minerals such as calcium, copper, and zinc may weaken the strength and elasticity of hoof tissues, making cows more susceptible to lameness [19]. At the same time, B-vitamins, as metabolic products of rumen microbes, are not only involved in energy metabolism but also play an important role in the keratinization process of the hooves [19]. Some studies have also indicated that specific fatty acids, such as linoleic acid and its metabolites, might influence hoof health by affecting the composition and metabolism of the rumen microbiota [19]. Although these studies have not fully elucidated the mechanisms, the results suggest that changes in the rumen microbiota could be a key factor in the occurrence of lameness. Furthermore, most of the current research focuses on severe lameness cases. However, a systematic study of mild cases could comprehensively reveal the development process of lameness and provide more meaningful insights for their prevention and treatment.

Based on these findings, this study hypothesizes that the composition of the rumen microbiota differs significantly between healthy cows and those with lameness and that certain key microbial species may play an important role in the development of lameness. To test this hypothesis, we compared the rumen microbiota composition and blood immune indicators of 11 cows with mild lameness and 10 healthy cows under consistent management conditions. By focusing on cows with mild lameness, this study aims to provide more meaningful insights into the mechanisms underlying the development of lameness and offer guidance for its prevention and treatment strategies.

## 2. Materials and Methods

### 2.1. Animals, Management, and Clinical Examination

This experiment was conducted at a commercial dairy farm in Shunyi District, Beijing, China, under conditions similar to previous studies [20]. All cows were managed with ad libitum access to feed and water and housed in ventilated free-stall barns with 120% bedding capacity relative to the herd size. The cows were fed a total mixed ration (TMR) formulated according to NRC (2001) requirements, provided three times daily (07:00, 14:30, and 22:00). The detailed composition of the TMR is provided in Table S1.

In this study, 182 cows (81 ± 14 days in milk) were monitored for one month by an experienced veterinarian. The health monitoring included daily observations of overall behavior, milk yield, and feed intake. Behavior assessments focused on identifying changes in posture, gait, and activity levels, such as prolonged lying time, reluctance to stand, or irregular walking patterns. Milk yield was recorded electronically during each milking session and reviewed for significant deviations from individual baseline levels. Feed intake was monitored by tracking the consumption of the TMR provided, with uneaten feed weighed and recorded daily. Additionally, weekly veterinary examinations were conducted to check for early signs of diseases, including detailed inspections of the hooves for swelling, lesions, or abnormalities; palpation of joints for signs of pain or inflammation; and overall assessments of body condition, hydration status, and rumen fill. After this monitoring period, a total of 21 cows were selected, including 10 healthy cows (BCS: 3.5 ± 0.27; age: 4.90 ± 0.81 years; DIM: 80 ± 2.36; parity: 2.90 ± 0.57; milk yield: 47.1 ± 7.7 kg) and 11 cows with mild lameness (BCS: 3.5 ± 0.39; age: 5.28 ± 0.55 years; DIM: 81 ± 2.48; parity: 3.19 ± 0.5; milk yield: 45.3 ± 6.2 kg). The cows were selected and grouped to ensure comparability between groups based on lactation stage, parity, and milk yield, minimizing potential confounding factors. Mild lameness was diagnosed based on a gait scoring system (5-point scale) [21]:

Score 1: Normal posture while standing and walking; steady gait.

Score 2: Straight back while standing but slightly arched back while walking; minor head tilt; slight gait abnormality.

Score 3: Arched back both while standing and walking; reduced stride length in one limb.

Score 4: Noticeable arched back while standing and walking; reliance on certain limbs or hooves for weight bearing.

Score 5: Severe arched back both while standing and walking; complete non-use of the affected hoof for weight bearing; difficulty standing, often recumbent.

Cows were classified into two groups based on their gait scores and health status. Mildly lame cows were those with a consistent gait score of 2 or 3 on two consecutive days, as assessed by an experienced veterinarian. These cows exhibited gait abnormalities but were excluded if they had evident hock injuries or secondary infections or swelling caused by external hoof damage, such as foreign body penetration or severe ulcers. Additionally, during the veterinarian’s weekly routine checkups, the mildly lame cows did not have concurrent diseases, such as mastitis or metabolic disorders. Metabolic disorders were determined based on the veterinarian’s assessment, which included monitoring blood BHB levels, milk production, and behavior. In contrast, healthy control cows consistently had a gait score of 1, indicating normal posture and gait, with no history of lameness, hoof damage, or systemic illness in the preceding 30 days.

### 2.2. Samples and Data Collection

After the cows for sampling were identified, rumen fluid and blood samples were collected the next morning before feeding. Rumen fluid was collected using a sterilized rumen tube and vacuum syringe. To avoid saliva contamination, the first 20% of the collected liquid was discarded. The pH of the rumen fluid was then measured using a portable pH meter (206-pH1, Testo, Shanghai, China). Finally, the samples were transferred to 1.5 mL cryovials and immediately stored in liquid nitrogen for subsequent amplicon analysis. Blood samples were collected from the caudal root vein into 10 mL tubes without anticoagulant. The samples were then centrifuged at 1500× *g* for 30 min at 4 °C (Tiangen OSE-MP25, Beijing, China) to separate the serum, which was subsequently stored in 1.5 mL centrifuge tubes. Body condition scores (BCS) were assessed by the experienced veterinarian using the method described by Edmonson et al. [22]. Milk production data were recorded and exported using the BouMatic systems (BouMatic, Madison, WI, USA).

### 2.3. Serum Inflammatory Factors and LPS Detection

The concentrations of serum tumor necrosis factor-α (TNF-α), interleukin-8 (IL-8), IL-6, IL-1, and lipopolysaccharide (LPS) were determined using respective Elisa kits (Nanjing Jian Cheng Bioengineering Institute, Nanjing, China).

### 2.4. DNA Extraction, Amplification, 16S rRNA Sequencing, and Bioinformatics Analysis

Microbial DNA was extracted from 21 rumen fluid samples using the PowerSoil DNA Isolation Kit (MoBio Laboratories, Carlsbad, CA, USA). DNA concentration and quality were assessed using a NanoDrop NC2000 spectrophotometer (Thermo Fisher Scientific). High-quality DNA samples underwent 16S rRNA sequencing using the universal primers 338F (ACTCCTACGGGAGGCAGCAG) and 806R (GGACTACHVGGGTWTCTAAT), targeting the V3–V4 region. Ten-base barcodes were added to the 5′ ends of primers for sample identification. PCR reactions were performed in a Mastercycler Gradient system (Eppendorf, Hamburg, Germany) with a total reaction volume of 25 μL, containing 12.5 μL 2× Taq PCR MasterMix, 3 μL BSA (2 ng/μL), 2 μL of each primer (5 μM), 2 μL template DNA, and 5.5 μL ddH_2_O. The PCR conditions were 95 °C for 5 min for initial denaturation, followed by 32 cycles of 95 °C for 45 s, 55 °C for 50 s, and 72 °C for 45 s, with a final extension at 72 °C for 10 min. Each sample was amplified in triplicate, and the PCR products were pooled to minimize bias. The pooled products were purified using the QIAquick Gel Extraction Kit (QIAGEN, Hilden, Germany) and quantified by real-time PCR. Final samples were sequenced on an Illumina MiSeq-PE300 platform (Illumina Inc., San Diego, CA, USA), generating 2 × 300 bp paired-end reads.

Raw reads were merged using Flash v1.2.0 [23] and demultiplexed based on unique barcodes. Reads were filtered to remove sequences <230 bp in length, with quality scores ≤20, ambiguous bases, or mismatched barcodes or primers. Filtered reads were clustered into operational taxonomic units (OTUs) at 97% similarity using USEARCH v8.1 [24], and representative sequences were taxonomically annotated using the SILVA ribosomal RNA database with the RDP classifier tool [25,26].

### 2.5. Downstream Analysis of 16S rRNA Sequencing Data and Statistical Analysis

The OTU data table was flattened based on the minimum sequence count per sample and analyzed using the TUTU Cloud platform (http://www.cloudtutu.com, accessed on 4 December 2024) for downstream analysis and visualization. Microbial richness and diversity were evaluated using the Chao1 and Shannon indices, and group differences were assessed with the Kruskal–Wallis test. Bray–Curtis dissimilarity matrices were used to analyze the rumen microbial community structure between groups, and differences were tested with PERMANOVA method. The difference analysis of phylum and family level differential bacteria was performed using Kruskal–Wallis test. For genera with relative abundance >0.001% and prevalence >50%, differential genera between groups were identified using the Kruskal–Wallis test and visualized with heatmaps. A co-occurrence network was constructed based on the top 80 genera using Spearman correlation analysis (R > 0.5, *p* < 0.05). The neutral community model (NCM) of rumen microbiota was analyzed using the flattened OTU table, as described by Sloan et al. [27]. Microbial functional features were predicted using PICRUSt2 (v2.4.1) based on 16S rRNA data. Functional differences between groups were analyzed at the L3 pathway level using STAMP v2.1.3, with significant metabolic pathways visualized in forest plots.

Statistical analyses of cow characteristics and blood indicators were performed using R software (v4.0.2). Normality was assessed using the Shapiro–Wilk test, and data not meeting normality assumptions were log-transformed. BCS and gait score data were analyzed using chi-squared tests, while other continuous variables were compared using independent-sample t-tests. Results were presented as mean ± standard deviation (SD). Statistical significance was set at *p* < 0.05, with 0.05 ≤ *p* < 0.1 considered a trend significance.

## 3. Results

### 3.1. Health Characteristics of Healthy and Lame Cows

**Figure 1:**
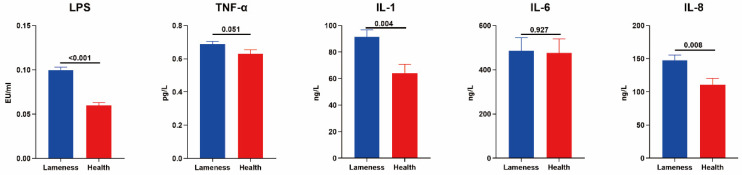
Blood LPS and inflammatory factors in two groups of cows.

As shown in Table 1, no significant differences were observed in BCS, age, lactation stage, parity, or milk yield between the two groups of cows. However, the rumen pH in the lame group was significantly lower than that in the healthy group (*p* < 0.05), while the gait score was significantly higher in the lame group (*p* < 0.05). In addition, concentrations of lipopolysaccharides (LPS) and inflammatory factors in blood were measured. The results indicated that the concentrations of LPS, IL-1, and IL-8 in the blood of the lame group were significantly higher than those in the healthy group (*p* < 0.05), with TNF showing a trend toward increased (0.05 ≤ *p* < 0.1). In contrast, no significant difference was observed in IL-6 levels between the two groups (Figure 1).

### 3.2. Microbial Diversity

**Figure 2:**
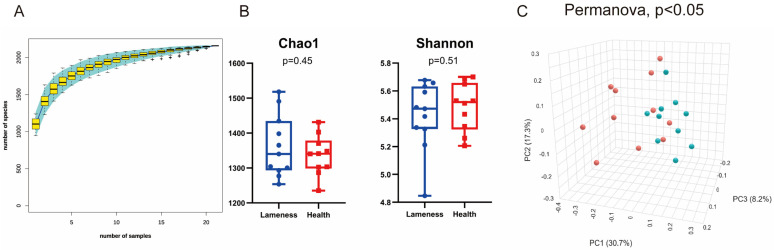
A comparison of rumen microbial diversity between lame and healthy cows. (**A**) Species accumulation curve of the samples; (**B**) a comparison of α-diversity between the two groups of cows; (**C**) a comparison of β-diversity between the two groups of cows; the red dots represent lameness group and green dots represent health group.

A total of 907,118 high-quality sequences were obtained from 21 rumen fluid samples, with an average of 43,196 ± 6162 sequences per sample. Species rarefaction curves flattened as sample size increased, indicating sufficient sequencing depth (Figure 2A).

To analyze differences in rumen microbiota between healthy and lame cows, microbial richness and diversity at the OTU level were evaluated using Chao1 and Shannon indices. No significant differences were found in Chao1 and Shannon indices between the two groups (0.1 < *p*, Figure 2B). However, principal coordinate analysis (PCoA) based on Bray–Curtis distances revealed significant separation of the microbial communities at the OTU level between the two groups (PERMANOVA, *p* < 0.05, Figure 2C), indicating significant differences in microbial community structure.

### 3.3. Microbial Composition

**Figure 3:**
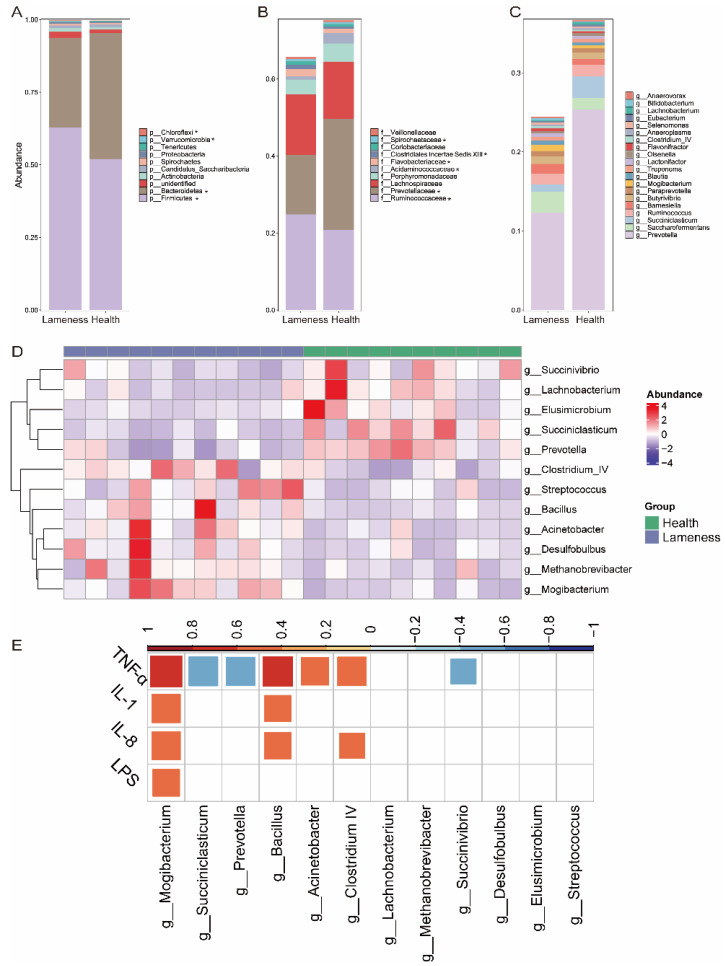
A comparison of microbial community composition between the two groups of cows. The stacked bar plots show the rumen composition of the two groups of cows at: (**A**) the phylum level, (**B**) the family level, and (**C**) the genus level; (**D**) heatmap showing the abundance of differentially abundant genera across different samples; (**E**) a correlation analysis between differential genera and differential blood parameters. * represents a significant difference between the two groups.

Microbial composition was analyzed at the phylum, family, and genus levels. At the phylum level, dominant bacteria in both groups included Firmicutes, Bacteroidetes, Actinobacteria, Candidatus, and Spirochaetes (Figure 3A). The healthy group showed significantly higher abundances of Bacteroidetes, while the lame group exhibited higher abundances of Firmicutes, Chloroflexi, and Verrucomicrobia.

At the family level, major families included *Ruminococcaceae*, *Prevotellaceae*, *Porphyromonadaceae*, and *Acidaminococcaceae*. *Prevotellaceae* and *Acidaminococcaceae* were significantly increased in the healthy group, whereas *Ruminococcaceae*, *Flavobacteriaceae*, *Clostridiales Incertae Sedis XIII*, and *Streptococcaceae* were significantly increased in the lame group (Figure 3B).

At the genus level, high-abundance genera included *Prevotella*, *Saccharofermentans*, *Succiniclasticum*, *Ruminococcus*, and *Butyrivibrio* (Figure 3C). Differential analysis showed that *Succinivibrio*, *Lachnobacterium*, *Elusimicrobium*, *Succiniclasticum*, and *Prevotella* were significantly increased in the healthy group, whereas *Clostridium IV*, *Streptococcus*, *Bacillus*, *Acinetobacter*, *Desulfobulbus*, *Methanobrevibacter*, and *Mogibacterium* were significantly increased in the lame group (Figure 3D). A correlation analysis revealed significant associations between concentrations of blood LPS, TNF, IL-1, and IL-8, and some differential genera (Figure 3E).

### 3.4. Microbial Interaction Networks and Community Modeling

**Figure 4:**
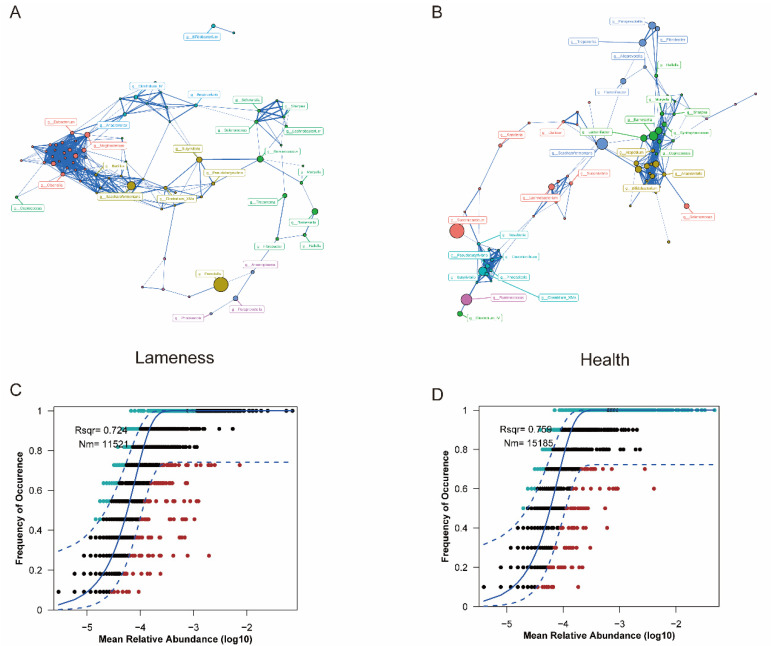
Microbial interaction analysis between the two groups of cows. Rumen microbial genus interaction network analysis of cows: (**A**) lame cows, (**B**) healthy cows; neutral community model analysis of rumen microbiota in cows based on OTUs: (**C**) lame cows, (**D**) healthy cows.

Interaction networks of the top 80 genera were constructed. Compared to the healthy cows, the lame group exhibited a more complex microbial interaction network (Figure 4A,B). Key genera such as *Mogibacterium, Clostridium,* and *Bacillus* were central nodes in the interaction network of the lame group, forming critical modules and driving complex relationships. In contrast, *Succiniclasticum, Succinivibrio,* and *Elusimicrobium* dominated the interaction network of the healthy group.

In addition, we evaluated the ecological structure of rumen microbial communities in each group using the NCM (Figure 4C,D). The results indicated that microbial ecosystems were predominantly governed by stochastic processes, with higher stochasticity observed in healthy cows compared to lame cows.

### 3.5. Microbial Functional Predictions

**Figure 5:**
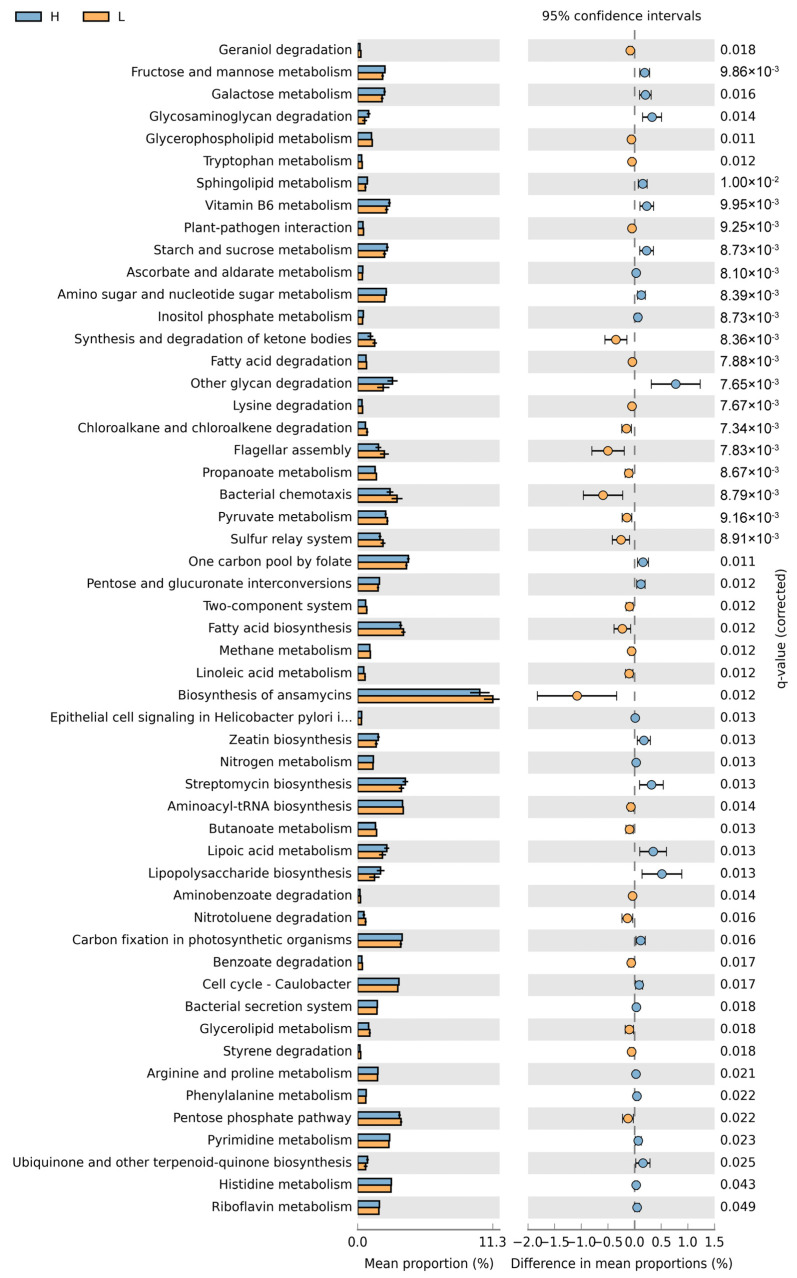
A comparison of the PICRUSt2 predicted pathways in the rumen fluid microbiota between the two groups of cows (only pathways with significant differences at L3 of Kegg are shown). H, health group; L, lameness group.

Functional prediction using PICRUSt2 revealed significant differences in microbial metabolic pathways between the two groups (Figure 5). Microbial functions related to glycosaminoglycan degradation, lipopolysaccharide biosynthesis, starch and sucrose metabolism, vitamin B6 metabolism, and nitrogen metabolism were significantly upregulated in the healthy group. In the lame group, microbial pathways involved in geraniol degradation, the synthesis and degradation of ketone bodies, flagellar assembly, bacterial chemotaxis, linoleic acid metabolism, and propanoate metabolism were significantly upregulated.

## 4. Discussion

Lameness is a prevalent disease in dairy cows, causing significant economic losses to farmers [4]. This study, for the first time, investigated the rumen microbiota of mildly lame cows compared to healthy cows under similar dietary, parity, age, and lactation stage conditions. The results revealed significant differences in the rumen microbiota between the two groups, suggesting that these microbial differences might be critical factors in the development of lameness. These findings provide novel insights into the relationship between rumen microbiota and lameness and may inform future diagnostic and therapeutic strategies.

The basic health parameters among the groups, such as BCS, age, lactation stage, and milk yield, showed no significant differences, eliminating the potential confounding effects of these factors. The rumen pH in the lameness group was significantly lower, and the average pH in lame cows was not below 5.6, indicating that they were not affected by rumen acidosis. This result further suggests a potential association between lameness and ruminal microbiota dysbiosis. The health of the rumen microbiome is crucial for maintaining rumen function and overall cow health. Rumen pH is typically regulated by various factors such as diet composition, water quality, and microbial balance [14]. A decrease in pH may result from the proliferation of harmful bacteria, leading to changes in fermentation products and subsequently affecting animal health. The elevated LPS levels in lame cows further support this hypothesis. LPS, components of the outer membrane of Gram-negative bacteria, are released upon bacterial death and can trigger inflammatory responses in the host [28]. This phenomenon may reflect an increase in Gram-negative bacteria within the rumen microbiome. LPS is released into the bloodstream through the outer membrane, activating the host immune system and inducing a systemic inflammatory response, which can exacerbate tissue damage and pathological processes. Cytokines, as important signaling molecules of the immune system, regulate inflammation and immune responses [29]. Elevated levels of TNF, IL-1, and IL-8 in lame cows indicate a systemic inflammatory state. TNF-α and IL-1 primarily function in acute inflammatory responses, activating immune cells and promoting local tissue damage to enhance inflammation [30,31]. IL-8, as a leukocyte chemokine, induces the recruitment of more leukocytes to the site of inflammation [32]. Although IL-6 is a pro-inflammatory cytokine [33], its blood levels did not show significant changes in this study, which does not mean it lacks a role in local inflammation. As a marker of chronic inflammation, IL-6 may not show a significant increase in whole blood, particularly in the case of mildly lame cattle. Future studies could further explore the expression of IL-6 in local tissues and its interactions with other inflammatory factors. This observation highlights the role of LPS elevation caused by microbial dysbiosis in the pathogenesis of lameness, suggesting that ruminal microbiota imbalance is not only closely associated with local inflammation but may also trigger a systemic inflammatory response through the interaction of LPS and cytokines. The interaction between ruminal microbiota imbalance and inflammation may play a key role in the development of lameness, providing valuable insights for future research and therapeutic strategies.

Although the Chao1 and Shannon indices showed no significant differences in microbial diversity between groups, PCoA analysis revealed clear differences in microbial community structure. This finding emphasizes that changes in microbial composition, rather than diversity, may more directly reflect the relationship between the rumen microbiome and lameness. Key microbial differences identified in the study include the enrichment of *Mogibacterium* in the lameness group. *Mogibacterium* is a Gram-positive anaerobic rod bacterium, previously isolated from the root canals of patients with periodontal disease, and associated with the induction of IL-8 and TNF in chronic bacterial osteomyelitis and osteonecrosis of the jaw [34,35,36]. Additionally, Liu et al. found an increased relative abundance of *Mogibacterium* in the rumen of lambs fed a high-concentrate diet, and this abundance was correlated with the expression of Toll-like receptor genes in the rumen epithelium [37]. These findings suggest that *Mogibacterium* may contribute to the pathogenesis of lameness by activating Toll-like receptor genes in the rumen, thereby promoting inflammation in hard tissues. In contrast, the health group showed an enrichment of *Succinivibrio* and *Succinimonas*, known for their efficient carbohydrate metabolism, which influence butyrate production—a metabolite with anti-inflammatory properties that supports gut barrier function and host immunity [38,39]. Although butyrate levels were not directly measured in this study, it may have a potential protective role in healthy cows.

The PICRUSt2 analysis further revealed significant differences in microbial functional pathways. In healthy cows, pathways related to carbohydrate metabolism, vitamin metabolism, and amino acid metabolism were upregulated, reflecting a more comprehensive and efficient metabolic capacity of the rumen microbiome, contributing to ecological balance and host health. In contrast, the rumen microbiome of lame cows showed an upregulation of pathways associated with cell motility (e.g., flagellar assembly, chemotaxis), metabolite degradation, and signaling pathways, suggesting microbial adaptation to ecological stress or competition. For example, enrichment in glycerolipid and linoleic acid metabolism may indicate microbial adaptation to nutritional stress, while these metabolic pathway changes might also significantly impact host inflammatory responses. Overall, these findings further support the central role of microbial dysbiosis in the pathogenesis of lameness, highlighting the need for future studies to focus on functional validation of key microbial groups and the dynamic changes in metabolic pathways.

We assessed the ecological processes of rumen microbiomes in lame and healthy cows using NCM. The results showed that deterministic processes dominated the rumen microbiome of lame cows, suggesting that strong external disturbances or adaptive pressures might lead to fixed and irreversible ecological trajectories. This determinism could arise from the microbiome response to ecological stress under pathological conditions, including rumen acidification, the activation of host immune responses, and competition among microbes driven by the proliferation of Gram-negative bacteria. In contrast, the rumen microbiome of healthy cows exhibited higher randomness, indicating a more resilient and stable ecosystem. Microbial ecosystems with high randomness typically show stronger resistance to disturbances, the ability to recover quickly after environmental changes, and the capacity to maintain functionality and diversity. Previous studies have linked high-grain diets to lameness in cows [17]; dietary factors were consistent across groups in this study, eliminating the potential impact of dietary interference. However, the sorting behavior of the cows may still be an important influencing factor [40]. Despite the same feeding conditions in this study, lame cows may have selectively consumed more grain in their diet, which could alter the composition of their ruminal microbiota. While we do not have direct evidence that sorting behavior is the sole cause of the microbiota differences, the significant decrease in rumen pH observed in the lameness group may indirectly indicate the impact of this behavior. Overall, apart from the ecological dynamics of the microbiota itself, the cows’ feeding behavior could be an important external factor contributing to the differences in ruminal microbiota, warranting further investigation.

This study highlights the potential link between lameness in cows and rumen microbial dysbiosis, suggesting that microbial imbalance may play a critical role in the pathogenesis of lameness by inducing host inflammatory responses. The interaction between microbial dysbiosis and inflammation might constitute a key mechanism underlying lameness. For instance, the deterministic microbiome in lame cows may exhibit lower functional diversity and metabolic flexibility, leading to the overproduction of pro-inflammatory metabolites such as LPS, which in turn triggers systemic inflammatory responses in the host. This mechanism further supports the causal role of the rumen microbiome in the development of lameness and provides an important theoretical basis for future research. In addition, to further elucidate the causal relationship between the rumen microbiome and lameness, future studies should integrate multi-omics approaches. For example, metabolomics could help analyze the dynamic changes in metabolites in rumen contents and host blood, identifying potential pro-inflammatory or anti-inflammatory molecular markers. Metagenomics could comprehensively reveal the functional potential of the rumen microbiome, identifying key microbial taxa and their metabolic pathways. By integrating these data, we could more precisely resolve the interaction networks between microbes and the host, uncovering their roles in the pathological progression of lameness. At the same time, probiotics (e.g., butyrate-producing strains) and feed additives (e.g., enzymes or prebiotics) could be used to restore rumen microbial balance, enhance the generation of anti-inflammatory metabolites, mitigate host inflammatory responses, and improve rumen metabolic efficiency. Additionally, microbiome-editing technologies, such as CRISPR-Cas systems, have the potential to precisely adjust the abundance and activity of key microbial taxa, fundamentally addressing ecological dysbiosis. Future research should also consider applying these strategies to different rearing environments and production conditions to evaluate their practical effectiveness and feasibility. This would not only aid in the development of preventive and therapeutic approaches for lameness but also improve overall rumen health, enhancing the productive performance of cows and supporting the sustainable development of the livestock industry.

## 5. Conclusions

This study comprehensively compared the rumen bacterial composition of lame and healthy dairy cows, revealing significant differences in microbial composition between the two groups. Notably, changes in the abundance of key bacterial taxa such as *Mogibacterium*, *Succiniclasticum*, *Succinivibrio*, and *Elusimicrobium* were closely associated with the occurrence of lameness. These findings provide important scientific evidence for the prevention and treatment of lameness in dairy cows. However, further research using multi-omics approaches is necessary to elucidate the specific roles of these key microbial taxa in the pathogenesis of lameness.

## Figures and Tables

**Table 1:** Health characteristics of healthy and lame cows (mean ± SD).

Items	Lameness (N = 11)	Health (N = 10)	*p*-Value
BCS ^1^	3.50 ± 0.39	3.50 ± 0.27	0.99
Age (years)	5.28 ± 0.55	4.9 ± 0.81	0.69
DIM ^2^ (days)	81 + 2.48	80 ± 2.36	0.72
Parity	3.19 ± 0.50	2.90 ± 0.57	0.69
Ruminal pH	5.89 ± 0.4	6.22 ± 0.3	<0.05
Milk yield (Kg/d)	45.3 ± 6.2	47.1 ± 7.7	0.84
Lameness scoring	2.27 ± 0.47	1	<0.05

^1^ BCS: body condition score; ^2^ DIM: days in milk.

## Data Availability

The 16S rDNA gene sequencing reads were deposited in the Genome Sequence Archive1 in the BIG Data Center under the accession number: PRJCA000874.

## Supplementary Materials

The following supporting information can be downloaded at: https://www.mdpi.com/article/10.3390/ani15040468/s1, Table S1. The composition and nutrition of TMR diet.
